# Computed tomographic angiography measures of coronary plaque in clinical trials: *opportunities and considerations to accelerate drug translation*

**DOI:** 10.3389/fcvm.2024.1359500

**Published:** 2024-03-04

**Authors:** N. Howden, K. Branch, P. Douglas, M. Gray, M. Budoff, M. Dewey, D. E. Newby, S. J. Nicholls, R. Blankstein, S. Fathieh, S. M. Grieve, G. A. Figtree

**Affiliations:** ^1^ Department of Cardiology, Royal North Shore Hospital, St Leonards, NSW, Australia; ^2^ Department of Cardiology, Gosford Hospital, Gosford, NSW, Australia; ^3^ Division of Cardiology, University of Washington, Seattle, WA, United States; ^4^ Duke Department of Medicine, The Duke University Medical Center, Durham, NC, United States; ^5^ Kolling Institute, University of Sydney, Sydney, NSW, Australia; ^6^ Department of Cardiology, Lundquist Institute, Torrance, CA, United States; ^7^ Department of Radiology, Charité – Universitätsmedizin Berlin, Humboldt-Universität zu Berlin, Freie Universität Berlin, Campus Mitte, Charitéplatz 1, Berlin, Germany; ^8^ Centre for Cardiovascular Science, University of Edinburgh, Edinburgh, United Kingdom; ^9^ Victorian Heart Institute, Monash University, Melbourne, VIC, Australia; ^10^ Departments of Medicine (Cardiovascular Division), Brigham and Women's Hospital, Harvard Medical School, Boston, MA, United States

**Keywords:** coronary plaque, coronary CT, drug development, atherosclerosis, CCTA

## Abstract

Atherosclerotic coronary artery disease (CAD) is the causal pathological process driving most major adverse cardiovascular events (MACE) worldwide. The complex development of atherosclerosis manifests as intimal plaque which occurs in the presence or absence of traditional risk factors. There are numerous effective medications for modifying CAD but new pharmacologic therapies require increasingly large and expensive cardiovascular outcome trials to assess their potential impact on MACE and to obtain regulatory approval. For many disease areas, nearly a half of drugs are approved by the U.S. Food & Drug Administration based on beneficial effects on surrogate endpoints. For cardiovascular disease, only low-density lipoprotein cholesterol and blood pressure are approved as surrogates for cardiovascular disease. Valid surrogates of CAD are urgently needed to facilitate robust evaluation of novel, beneficial treatments and inspire investment. Fortunately, advances in non-invasive imaging offer new opportunity for accelerating CAD drug development. Coronary computed tomography angiography (CCTA) is the most advanced candidate, with the ability to measure accurately and reproducibly characterize the underlying causal disease itself. Indeed, favourable changes in plaque burden have been shown to be associated with improved outcomes, and CCTA may have a unique role as an effective surrogate endpoint for therapies that are designed to improve CAD outcomes. CCTA also has the potential to de-risk clinical endpoint-based trials both financially and by enrichment of participants at higher likelihood of MACE. Furthermore, total non-calcified, and high-risk plaque volume, and their change over time, provide a causally linked measure of coronary artery disease which is inextricably linked to MACE, and represents a robust surrogate imaging biomarker with potential to be endorsed by regulatory authorities. Global consensus on specific imaging endpoints and protocols for optimal clinical trial design is essential as we work towards a rigorous, sustainable and staged pathway for new CAD therapies.

## Introduction

Atherosclerotic coronary artery disease (CAD) is the causal pathological process driving major adverse cardiovascular events (MACE) worldwide, and is responsible for over 9 million annual deaths (1). Identifying and treating standard modifiable cardiovascular risk factors (SMuRFs)—including elevated low-density lipoprotein (LDL) cholesterol, blood pressure (BP), smoking, and diabetes mellitus—has been an essential and effective component in reducing major adverse clinical events (MACE) for both primary and secondary cardiovascular prevention over the last decades. However, persistent risk for MACE and the recent flattening in rates of CAD mortality around the globe are concerning (2, 3). New mechanisms explaining individual CAD susceptibility and resilience, above and beyond these risk factors, are emerging through both candidate and unbiased discovery approaches with potential application into novel drug targets (4).

Clinical trials serve as indispensable tools to assess the safety and efficacy of novel interventions. The choice of appropriate reliable endpoints is paramount for the success of such trials, enabling accurate evaluation of therapeutic interventions and facilitating their translation into clinical practice. Traditionally, a composite endpoint of MACE (typically consisting of death or cardiovascular death, myocardial infarction, and stroke) has been used for phase 3 trials of coronary artery disease to gain regulatory approval. While this is highly relevant to both patients and health systems, it requires very large patient cohorts (typically more than 10,000) and years of data collection to detect potential therapeutic benefits. The immense commitment and cost required for such a trial is a major disincentive for novel approaches and less common disease states. The average cost for new CVD drug development is around USD$1.1 billion, with costs continuing to escalate over time (5). Thus, despite the substantial unmet need and size of the CAD market, the reliance on clinical endpoint-based trial pathways can turn industry away from risky financial investment into therapeutics which act via novel mechanisms.

Despite the well-established direct causal role of CAD in myocardial infarction (MI), there are currently no approved CAD imaging biomarkers for use as a surrogate clinical trial endpoints. The more traditional cardiovascular risk factors of LDL cholesterol and BP are recognised surrogate trial endpoints by the US Food and Drug Authority and can be used for drug approval. The need for CAD drug therapies that target pathophysiology beyond traditional risk factors and mechanisms is highlighted by the increasingly recognised importance of inflammatory, social and environmental risks and by the emerging group of patients who develop CAD in the absence of standard modifiable cardiovascular risk factors. The latter (termed “SMuRFless”), comprising up to 10–27 percent of patients with their first incident myocardial infarction (6, 7). Plaque can be detected and tracked in these patients using CCTA but given they lack standard risk factors, typical surrogates such as LDL and blood pressure cannot be utilised in this special population which then leads to their exclusion from trials and paucity of evidence for therapeutics in this group.

The need for improved efficiency for clinical translation without loss of rigor has led the U.S. Food & Drug Administration (FDA) to participate in multi-disciplinary efforts with many other disease fields to develop surrogate endpoints. Between 2010 and 2012, 45 percent of drugs were approved based on surrogates (8). In the quest for robust clinical trial endpoints, the utilisation of non-invasive imaging modalities of CAD has emerged as an approach to provide safe, accurate, and rapid assessment of therapeutic efficacy. Among these modalities, coronary computed tomography angiography (CCTA) is the most widely available with robust ability to visualize and measure coronary artery atherosclerosis with high spatial resolution and accuracy. Studies have demonstrated the ability of conventional CCTA to measure qualitatively and quantitatively pathophysiological features of CAD plaque in a manner similar to intravascular ultrasound and post-mortem histology (9–11). Recent advances in CT scanner and post-acquisition analysis, including application of artificial intelligence, have improved quantitation of non-calcified plaque and low-attenuation plaque, the latter of which is thought to be metabolically active component (12). Several studies have shown that improvements in CAD plaque morphology appear to be associated with reductions of MACE risk, but at present, this evidence is insufficient for application in regulatory approval. For acceptance as a surrogate measure o drug development tool, it is essential that academic clinicians engage with both the pharmaceutical industry and regulatory authorities to develop an evidence base and consensus on specific CT-based measures that most closely predict the effect of intervention on MACE (10).

The CAD Frontiers *Atherosclerosis CT Imaging Outcome Consortium: Accelerating Atherosclerosis Drug Development* group (ACTION A2D2) has been formed to facilitate a stepwise multi-disciplinary stakeholder engagement process to develop consensus on specific candidate CCTA plaque measurements that relate to the causal pathway for MACE. The Consortium includes leading clinical trialists, clinicians, industry, regulatory authorities (including FDA), and patients. Ultimately, the ACTION A2D2 group seeks to explore the use of CCTA measurements as a diagnostic biomarker for enrichment of clinical trial patient selection, a prognostic biomarker for pharmacodynamic engagement, and a predictive biomarker as a clinical trial surrogate endpoint. If successful, reductions in the size and length of the required clinical trials with CCTA plaque endpoints have the potential to de-risk the clinical development pathway for novel CAD therapies, accelerate candidate drugs into human testing, and prioritize candidate drugs for clinical cardiovascular outcome trials (CVOTs). In this paper, we discuss the evidence for CCTA to fulfill this role, and the key next steps required to meet the rigorous standards to be accepted as a valid surrogate for drug trials.

## Advantages of coronary CT angiography in assessment of coronary disease: lessons from the clinic relevant to trials

CCTA has become the preferred first-line investigation for troponin negative acute chest pain and in many cases as a risk stratification tool in CAD, with Level 1A endorsement from the American Heart Association (AHA) and American College of Cardiology (ACC), the European Society of Cardiology, and National Institute for Health and Care Excellence guidelines (13). European Society of Cardiology (ESC) 2019 guidelines on stable coronary disease gave CCTA a level 1B recommendation (14). Imaging techniques and technology have developed rapidly over the past few decades from the simple calcium score to coronary luminal stenosis to the modern detection of detailed plaque features. The ability to detect obstructive CAD (greater than 50% stenosis by invasive angiography) is high with a sensitivity of 95.2% and specificity of 79.2%, improving greatly over time (11, 12, 15). It is the only diagnostic test for CAD shown to guide clinical care in a manner that reduces heart attacks, where the CCTA group demonstrated lower rate of 5-year CAD mortality or non-fatal MI (2.3% vs. 3.9%) primarily driven by a reduction in risk of non-fatal MI (HR 0.60) in the SCOT-HEART Study (16). Furthermore, CCTA measures have proven to be superior risk predictors than ischaemia testing in the large PROMISE, SCOT-HEART and ISCHAEMIA studies (16–18). The DISCHARGE trial showed that CT reduces major procedure-related complications (HR 0.26) and possibly reduces major adverse cardiovascular events (HR 0.7) compared with management following invasive coronary angiography (19). Importantly, multiple studies have shown an equivalent risk for patients with extensive non-obstructive CAD (<50% stenosis) as compared to those with one vessel obstructive CAD (20, 21). Individuals with any non-obstructive plaque have been shown to have higher mortality than those with normal coronary arteries (22). These findings demonstrate the value-add of non-invasive CCTA imaging for MACE risk assessment, particularly as CCTA is the only imaging test that can detect non-obstructive CAD. The detection of non-obstructive CAD is critical in patients with no other evidence of atherosclerosis who would otherwise not warrant the same aggressive prevention therapies and the detection of these patients through CCTA would allow their inclusion in trials after detection in clinic.

### Plaque phenotyping

An important CCTA feature relevant for application in clinical trials is the ability to analyse and to quantify specific elements of coronary plaque. Through the use of Hounsfield Unit assessments, CT imaging can detect plaques at various stages of atherosclerosis including calcified plaque, fibrous and fibrofatty plaque, and earlier and more metabolically active lipid-rich plaques. Additional data can include markers such as pericoronary and epicardial fat. Several high-risk plaque (HRP) features are associated with higher rates of clinical events (23), such as low-attenuation plaque, spotty calcification, positive remodelling, and the so-called “napkin ring sign”. This is particularly valuable if the imaging endpoint is used as an enrichment biomarker for MACE-based studies, or as a pharmacodynamic response biomarker to assess the effect of therapy. Volumetric quantitation and differentiation of plaque subtypes are available with intra-coronary imaging modalities of intravascular ultrasound and optical coherence tomography, but the invasive nature of needing to cannulate and image from within a coronary artery limits wide applicability. These interventional techniques are also limited to larger more proximal vessels and can be difficult to pass through severely stenotic lesions. Fortunately, accuracy of CCTA to analyse plaque morphology compares favourably with these invasive techniques (24–26). However, for purposes of measuring potential benefits of new therapeutic interventions targeting plaque, the most consistent CCTA endpoints involve the change in volume or percentage of non-calcified plaque (Table 1) with the additional advantage of being a continuous quantitative variable. The advantage of non-calcified plaque over total plaque burden is due to the observed increase in coronary calcification with stabilisation or regression of other types of plaque. This can result in an apparent neutral change or even an increase in total plaque volume despite overall plaque stabilisation. Furthermore, non-calcified plaque is also associated with higher risk plaques, and is plaque that is more likely to be modified by medical therapy.

**Table 1:** Current trials with CCTA endpoints demonstrating change in plaque morphology and volume over time with therapies.

Study	Design	Intervention	Inclusion and exclusion criteria	Plaque analysis	Outcome measures	Result
Auscher et al. (27)	Design: Randomised, open label, control trial Total N: Mean follow up: 12 month	Treatment (Tx): Intensive statin therapy Control: Standard of care (SOC) statin therapy	Inclusion: – Post MI (NSTEMI or STEMI) Exclusion: – Ongoing high intensity statin therapy – Contraindication to statins – Prior or planned CABG – Contrast allergy – Cr >120 mmol/L. Non-sinus rhythm	Semi-automated plaque analysis software	Primary: – Total Plaque volume (mm³) Secondary: – Plaque composition Plaque vulnerability	Primary endpoint change: – Total plaque volume, Tx 43.5 vs. SOC 19.1, *p* = 0.57 Secondary Endpoint change: – Total dense calcium volume, Tx 11.1 vs. SOC −0.4, *p* ≤ 0.001 – Total fibrotic volume, Tx −3.5 vs. SOC −1.1, *p* = 0.94 – Total fibrofatty volume, Tx −3.5 vs. SOC −1.1, *p* = 0.29 – Total necrotic core volume, Tx 26.8 vs. SOC 25.2, *p* = 0.94 **Only statistically significant increase in calcific plaque volume with statin use**.
Li et al. (28)	Design: Multicentre prostpective observational study Total N: Mean follow up: 18 month	Group 1: Intensive dose statin (IS) Group 2: Moderate dose statin (MS) Group 3: No statin (NS)	Inclusion: – Age 18–80 – Single measurable, non-obstructive, non-calcified lesion on CTCA Exclusion: – Poor image quality – Prior statin use	Semi-automated plaque analysis software	Primary: – Total Plaque volume (mm³) – LAP volume (mm³) – Percentage plaque volume (%)	Primary endpoint change: – Total plaque volume, IS −16.4 vs. MS −0.1 vs. NS 12.3, *p* ≤ 0.001 – LAP volumes, IS −7.1 vs. MS −2.8 vs. NS 0.9, *p* ≤ 0.001 – Percentage plaque volume, IS −6.2 vs. MS −1.8 vs. NS 2.5, *p* ≤ 0.001 **Moderate and high intensity statins decrease total plaque volume, LAP volume and percentage plaque volume**.
Noguchi et al. (29)	Design: Prospective open-label, propensity score matched study Total N: 132 Mean follow up: 12 month	Treatment (Tx): Pitavastatin to aim LDL <80 mg/dl Control: Propensity match controls (PMC) with dietary intervention only	Inclusion: – Presence of coronary artery plaque – Exclusion: – Prior statin use – Scheduled PCI – eGFR <60 – Severely calcified lesion on CTCA – Unstable angina – Poor CMR or CTCA image quality	Semi-automated plaque analysis software	Primary: – MRI endpoint Secondary: – Total plaque volume (mm³) – LAP volume (mm³) – Percent total plaque volume (%) – Percent LAP volume (%) – Remodelling index (RI)	Primary endpoint change: – MRI endpoint Secondary Endpoint change: – Total plaque volume (mm³), Tx −5.0 vs. PMC 12.4, *p* = 0.028 – LAP volume (mm³), Tx −12.8 vs. PMC 8.3, *p* = 0.004 – Percent total plaque volume (%), Tx −4.6 vs. PMC 3.1, *p* = 0.108 – Percent LAP volume (%), Tx −11 vs. PMC 9.9, *p* ≤ 0.001 Remodelling index (RI), Tx −2.4 vs. PMC 1.7, *p* = 0.138 **Pitavastatin decreases LAP volume and percent LAP volume.**
Zeb et al. (30)	Design: Retrospective observational study Total N: 100 Median follow up: 406 days	Group 1: Statin users (St) Group 2: Non-statin users (NSU)	Inclusion: – 2 consecutive CCTA at least 1 year apart Exclusion: – Poor image quality – Interim coronary revascularisation	Semi-automated plaque analysis software	Primary: – LAP volume (mm³) – Non-calcified plaque volume (mm³) – Calcified plaque volume (mm³)	Primary endpoint change (Adjusted from regression): – LAP volume (mm³), St −18.1 vs. NSU 0, *p *≤ 0.001 – Non-calcified plaque volume (mm³), St −101.7 vs. NSU 0, *p *≤ 0.001 – Calcified plaque volume (mm³), St 41.5 vs. NSU 0, *p* = 0.245 **Statins reduce LAP volume and non-calcified plaque volume.**
Shin et al. (31)	Design: Multi centre observational study Total N: 147 Mean follow up: 24 months	Group 1: Follow up LDL-C < 70 mg/dl Group 2: Follow up LDL-C ≥ 70 mg/dl	Inclusion: – LDL lab data available within 1 month of first CCTA Exclusion: – No plaque – CABG – Poor image quality – Excluded stents from per-vessel analysis	Semi-automated plaque analysis software	Primary: – Change in plaque volume (mm³) – Annual change in plaque volume (mm³)	Primary endpoint change: – Change in plaque volume (mm³), Grp1 12.7 vs. Grp2 41.8, *p* = 0.009 – Annual change in plaque volume (mm³), Grp1 4.6 vs. Grp2 14.5, *p* = 0.015 **Achievement of LDL-C < 70 mg/dl is associated with reduced rate and volume of plaque progression.**
Budoff et al. (32)	Design: Randomised,double blind, placebo controlled trial Total N: 170 Mean follow up: 12 months	Treatment (Tx): Testosterone supplementation Control: Placebo (P)	Inclusion: – Age >64 – Serum testosterone <275 ng/dl – Sexual dysfunction – Physical dysfunction – Reduced vitality Exclusion: – High risk prostate Ca – CVA/MI in last 3 months – SBP >160 – DBP >100 – eGFR <60 – allergy to iodine contrast – weight >136 kg – arrhythmia – CABG	Semi-automated plaque analysis software	Primary: – Non-calcified plaque volume (mm³) Secondary: – Total plaque volume (mm³) – Low attenuation plaque volume (mm³) – Fibrous-fatty plaque volume (mm³) – Fibrous plaque volume (mm³) – Dense calcified plaque volume (mm³)	Primary endpoint change: – Non-calcified plaque volume (mm³), Tx 54 vs. P 14, *p* = 0.003 Secondary Endpoint change: – Total plaque volume (mm³), Tx 75 vs. P 28, *p* = 0.006 – Low attenuation plaque volume (mm³), Tx 8 vs. P 3, *p* = 0.14 – Fibrous-fatty plaque volume (mm³), Tx 12 vs. P 2, *p* = 0.11 – Fibrous plaque volume (mm³), Tx 31 vs. P 7, *p* = 0.01 – Dense calcified plaque volume (mm³), Tx 17 vs. P 11, *p* = 0.51 **Testosterone supplementation was associated with increased volume non-calcified plaque, total plaque volume, and fibrous plaque volume.**
Vaidya et al. (33)	Design: Prospective, open label, non-randomised observational study Total N: 80 Median follow up: 12.6 months	Treatment (Tx): Colchicine with OMT Control: OMT alone (OMT)	Inclusion: – - ACS &lt;1month ago Exclusion: – CABG – Allergy/hypersensitivity to colchicine – Existing colchicine therapy – Severe liver disease – Renal insufficiency – CYP3A4 inhibitors – Haematological malignancy – Thrombocytopenia – Leucopenia – Chronic inflammatory bowel disease – Pregnancy or risk of pregnancy – Lactating women	Semi-automated plaque analysis software	Primary: – Change in low-attenuation plaque volume (mm³) Secondary: – Total plaque volume(mm³) – Non-calcified plaque volume (mm³) – Dense calcified plaque volume (mm³) – Remodelling index (RI)	Primary endpoint change: – Change in low-attenuation plaque volume (mm³), Tx −15.9 vs. OMT −6.6, *p* = 0.008 Secondary Endpoint change: – Total plaque volume(mm³), Tx −42.3 vs. OMT −26.4, *p* = 0.10 – Non-calcified plaque volume (mm³), Tx −26.3 vs. OMT −18.2, *p* = 0.21 – Dense calcified plaque volume (mm³), Tx −0.1 vs. OMT −1.6, *p* = 0.47 – Remodelling index (RI), Tx −0.04 vs. OMT −0.01, *p* = 0.16 **Colchicine reduces low-attenuation plaque volume.**
Budoff et al. (34)	Design: Multi centre randomized double blind placebo controlled trial Total N: 80 Mean follow up: 18 months	Treatment (Tx): Icosapent ethyl (IPE) 4 g/day Control: Mineral oil placebo (P)	Inclusion: – Age 30–85 – Known atherosclerosis (>20% in at least 1 artery via CTCA or angio) – TG 135–499 mg/dl – LDL-C 40–115 mg/dl – Stable on statin therapy >4 weeks Exclusion: – Not on statins – Contrast allergy	Semi-automated plaque analysis software	Primary: – Low attenuation plaque volume (% *Δ*) Secondary: – Total plaque volume (% *Δ*) – Total non-calcified plaque volume (% *Δ*) – Fibrofatty plaque volume (% *Δ*) – Fibrous plaque volume (% *Δ*) – Calcified plaque volume (% *Δ*)	Primary endpoint change: – Low attenuation plaque volume (mm³), Tx −17 vs. P 109, *p* = 0.0061 Secondary Endpoint change: – Total plaque volume (mm³), Tx −9 vs. P 11, *p* = 0.0019 – Total non-calcified plaque volume (mm³), Tx −19 vs. P 9, *p* = 0.0005 – Fibrofatty plaque volume (mm³), Tx −34 vs. P 32, *p* = 0.0002 – Fibrous plaque volume (mm³), Tx −20 vs. P 1, *p* = 0.0012 – Calcified plaque volume (mm³), Tx −1 vs. P 15, *p* = 0.531 **IPE was associated with reduced LAP volume, total plaque volume, non-calcified plaque volume, fibro-fatty and fibrous plaque volume**.
Hirai et al. (35)	Design: Single centre retrospective comparative study Total N: 148 Mean follow up: 6 months	Treatment (Tx): 140 mg every 2 weeks Control: Standard of care (SOC) with statins	Inclusion: – Age >20 – 1 or more vulnerable plaque on CTCA, <50 HU within region remodel index >1.1 – Treatment with evolocumab >6 months – Statin >12 months before commence evolocumab – Carotid maximum initima-media thickness (IMT)change >0.0 mm/yr Exclusion: – Dialysis	Manual plaque analysis	Primary: – change in HU of vulnerable (LAP) plaque Secondary: – Size of vulnerable plaques (Remodelling index) – Percent stenosis at vulnerable plaque (%)	Primary endpoint change: – HU of vulnerable (LAP) plaque, Tx 45.8 vs. SOC 0 Secondary Endpoint change: – Size of vulnerable plaques (Remodelling index), Tx −0.10 vs. SOC −0.01 – Percent stenosis at vulnerable plaque (%), Tx −5.8 vs. SOC 4.7 **Groups not directly compared. Evolocumab increases the HU (stablises) vulnerable plaque within a 6 month time period (*p* ≤ 0.001).**
Inoue et al. (36)	Design: Prospective, non-randomised study. Total N: 32 Median follow up: 12 months	Treatment (Tx): Fluvastatin 20 mg Control: No statins (NS)	Inclusion: – Statin naïve Exclusion: – Heavily calcified lesions – Lesions with >75% stenosis – Plaques with prior PCI	Semi-automated plaque analysis software	Primary: – Total Plaque volume (mm³) Secondary: – Low attenuation plaque volume (mm³) – Lumen volume (mm³) – Remodelling index (RI)	Primary endpoint change: – Total Plaque volume (mm³), Tx −15.9 vs. NS 4, *p* = 0.01 Secondary Endpoint change: – Low attenuation plaque volume (mm³), Tx −3.6 vs. NS 0.2, *p* ≤ 0.01 – Lumen volume (mm³), Tx 1.3 vs. NS −5.5, *p* = 0.24 – Remodelling index (RI), Tx −1.7 vs. NS −0.3, *p* = 0.53 **Statin therapy decreased total plaque volume and low-attenuation plaque volume.**
Lee et al. (37)	Design: Observational study Total N: 1,255 Minimum follow up: 2 years	Group 1: Statin naive (SN) Group 2: Taking statins (TS)	Inclusion: – Suspected or known CAD undergoing CTCA – Included patients who had events between CTCA 1 and 2 Exclusion: – No CAD at baseline – No follow up CT – MI or revascularisation prior to CT – Patients without information on statin use prior to initial CT – Discontinued statin use after CTCA 1 – Poor quality scan	Semi-automated plaque analysis software	Primary: – Annualised within lesion change in percent atheromatous volume (PAV) (%/yr) Secondary: – Plaque composition, change in calcified PAV, Non-calcified PAV, Fibrous PAV, Fibro fatty PAV, Low attenuation PAV (%/yr) – development of HRP features (no#) – percent diameter of stenosis (%) – development of obstructive lesions (no#)	Primary endpoint change: – Change in PAV, SN 2.04 vs. TS 1.76, *p* = 0.002 Secondary Endpoint change: – Calcified PAV, SN 0.98 vs. TS 1.27, *p* ≤ 0.001 – Non-calcified PAV, SN 1.06 vs. TS 0.49, *p *≤ 0.001 – Fibrous PAV, SN 0.89 vs. TS 0.53, *p *≤ 0.001 – Fibro-fatty PAV, SN 0.16vs TS −0.03, *p* ≤ 0.001 – Low attenuation PAV, SN 0.01 vs. TS 0.00, *p* = 0.202 – New HRP, SN 62 vs. TS 86, *p* = 0.306 – diameter of stenosis, SN 6.6 vs. TS 5.7, *p* ≤ 0.001 – obstructive lesions, SN 11 vs. TS 36, *p* = 0.127 **Significant change in PAV, calcified PAV, non-calcified PAV, Fibrous PAV, Fibro-fatty PAV and diameter of stenosis.**
Lo et al. (38)	Design: Randomised, double blind, placebo controlled trial Total N: 40 Mean follow up: 12 months	Treatment (Tx): Atorvastatin 20–40 mg daily Control: Placebo (P)	Inclusion: – HIV positive – No history of CVD or Cardiac Symptoms – Plaque on CCTA – No stenosis >70% LM or >50% other coronary arteries – 18–60 yo – on Anti retroviral therapy – LDL 1.81–3.37 mmol/l Exclusion: – Contraindication to statin therapy – LFT derangement at baseline – Acute infective illness – Weight >136 kg – Iodine allergy	Semi-automated plaque analysis software	Primary: – FDG-PET endpoint Secondary: – Non-calcified plaque volume (mm³) – Total plaque volume (mm³) – Calcium score (Agatston) – Low attenuation plaque (no#) – Remodelling index >1.05 (no#) – Spotty calcification (no#)	Primary endpoint change: – Non-CT endpoint Secondary Endpoint change: – Non-calcified plaque volume, Tx −8.2 vs. 6.7, *p* = 0.03 – Total plaque volume, Tx −0.8 vs. P 12, *p* = 0.02 – Calcium score, Tx 0.9 vs. P 1.7, *p* = 0.74 – Low attenuation plaque (no#), Tx −1.2 vs. P 0.4, *p* = 0.03 – Remodelling index >1.05 (no#), Tx −0.2 vs. P 0.4, *p* = 0.04 – Spotty calcification (no#), Tx 0.0 vs. P −0.2, *p* = 0.25 **Significant change in non-calcified plaque volume and total plaque volume**.
Matsumoto et al. (39)	Design: Placebo controlled, double blinded trial Total N: 55 Mean follow up: 12 months	Treatment (Tx): Aged garlic extract 305 g Control: Placebo (P)	Inclusion: – 40–75 yo – 2 components of metabolic syndrome – 10 year Framingham risk score 6%–20% Exclusion: – Hypersensitivity to garlic – Renal impairment – Class II-IV heart failure – TG >400 mg/dl – Diabetes – Smoker	Semi-automated plaque analysis software	Primary: – Total plaque volume percentage (%) – Non-calcified plaque (%) – Low attenuation plaque (%) – Dense calcium (%)	Primary endpoint change: – Total plaque volume percentage (%), Tx 0.3 vs. P 1.6, *p* = 0.06 – Non-calcified plaque (%), Tx 0.2 vs. P 1.4, *p* = 0.09 – Low attenuation plaque (%), Tx −1.5 vs. P 0.2, *p* = 0.008 – Dense calcium (%), Tx 0.2 vs. P 0.2, *p* = 0.92 **Statistically significant changes in low-attenuation plaque percentage.**
Shaikh et al. (40)	Design: Single-center, randomized, placebo-controlled, double-blind trial Total N: 80 Mean follow up: 12 months	Treatment (Tx): Aged garlic extract 2,400 mg Control: Placebo (P)	Inclusion: – 30–75 yo – T2DM – HbA1c > 6.5% – Fasting BSL >125 mg/dl Exclusion: – weight >136 kg – hypersensitivity to garlic – bleeding disorder – known history of CAD, MI, stroke, arrythmia in last 6 months – NYHA HF II–IV – Renal impairment	Semi-automated plaque analysis software	Primary: – Rate of change in coronary plaque volume (*Δ*%) Secondary: – Dense calcium plaque (*Δ*%) – Fibro-fatty plaque (*Δ*%) – Fibrous plaque (*Δ*%) – Low-attenuation plaque (*Δ*%) – Total non-calcified plaque (*Δ*%)	Primary: – Rate of change in coronary plaque volume (*Δ*%), Tx 19 vs. P 55, *p* = 0.50 Secondary: – Dense calcium plaque (*Δ*%), Tx 69 vs. P 33, *p* = 0.82 – Fibro-fatty plaque (*Δ*%), Tx −35 vs. P 34, *p* = 0.28 – Fibrous plaque (*Δ*%), Tx 25 vs. P 10, *p* = 0.60 – Low-attenuation plaque (*Δ*%), Tx −29 vs. P 57, *p* = 0.04 – Total non-calcified plaque (*Δ*%), Tx 29 vs. P 81, *p* = 0.62 **Significant result in the rate of change in low attenuation plaque volume.**
Lu et. al. (41)	Design: Single-center, randomized, placebo-controlled, double-blind trial Total N: 804 Mean follow up: 24 months	Treatment (Tx): Pitavastatin Control: Placebo (P)	Inclusion: – 40–75 yo – HIV infection – stable anti-retroviral therapy – low to moderate risk of CVD Exclusion: – previous statin use – known atherosclerotic disease	Semi-automated plaque analysis software	Primary: – Change in non-calcified plaque volume (mm³) Secondary: – Low attenuation plaque volume (mm³)	Primary: – Change in non-calcified plaque volume (mm³), Tx −1.7 vs. P 2.62, *p* = 0.044 Secondary: – Low attenuation plaque volume (mm³), Tx −0.9 vs. P −0.1, *p* = 0.026 **Significant change in non-calcified plaque and low attenuation plaque volumes.**

CABG, coronary atery bypass graft; Cr, creatinine; CMR, cardiac magnetic resonance imaging; Ca, cancer; SBP, systolic blood pressure; DBP, diastolic blood pressure; TG, triglyceride; HU, Hounsfield units; CVD, Cardiovascular disease; LM - left main coronary artery; LFT, liver function tests; T2DM, type 2 diabetes mellitus; BSL, blood sugar level; NYHA, new York heart association; HIV, human immunodeficiency virus.

Advancements in CT hardware and software have improved spatial and temporal resolution to allow robust plaque analysis. Newer technologies, including photon-counting CT detectors, can overcome artefacts related to coronary calcification, having the potential to improve the accuracy and reproducibility of measures of non-calcified plaque components relevant for optimising clinical trials (42). In addition, semi-automated and automated systems have dramatically improved the speed and accuracy of plaque assessment, requiring only minor adjustments from human observers (43, 44). Post-acquisition analyses are also able to measure altered attenuation of peri-coronary adipose tissue (fat attenuation index or FAI) and have potential as incremental or standalone surrogate endpoints for clinical trials in the future (45). FAI has been demonstrated in the large CRISP-CT observational study to be associated with increased mortality however currently is not in widespread use due to the increased need for post processing and proprietary software (46).

#### Coronary CT angiography endpoints for enrichment

As discussed above, there is a substantial body of evidence for the prognostic value of CCTA measures of coronary plaque, such as total plaque volume, non-calcified plaque volume, presence of HRP features, and radiomic features. The direct relationship to the causal pathology of MACE and MI and this strong prognostic ability make these plaque measures valuable as potential enrichment biomarkers to identify higher risk patients for MACE. This is a recognised step for biomarker recognition and approval by the Food and Drug Administration as a drug development tool. In particular, establishing this link can allow for moving beyond enrichment based on well-understood classical cardiovascular risk factors, to enrichment based on the presence and make up of the actual disease that is being treated, CAD (Table 2). The strength of these studies is already high and may be suitable for a formal submission for regulatory approval.

**Table 2:** Evidence based enrichment outcomes from coronary CT angiography with the relevant features, findings in studies which support their use as enrichment outcomes, and examples of their appearance on CT or in post-processed images (23, 37, 46–49).

CT derived coronary features	Findings	
Total Plaque Volume	Baseline plaque volume was the most important predictor for lesions developing into obstructive lesions.	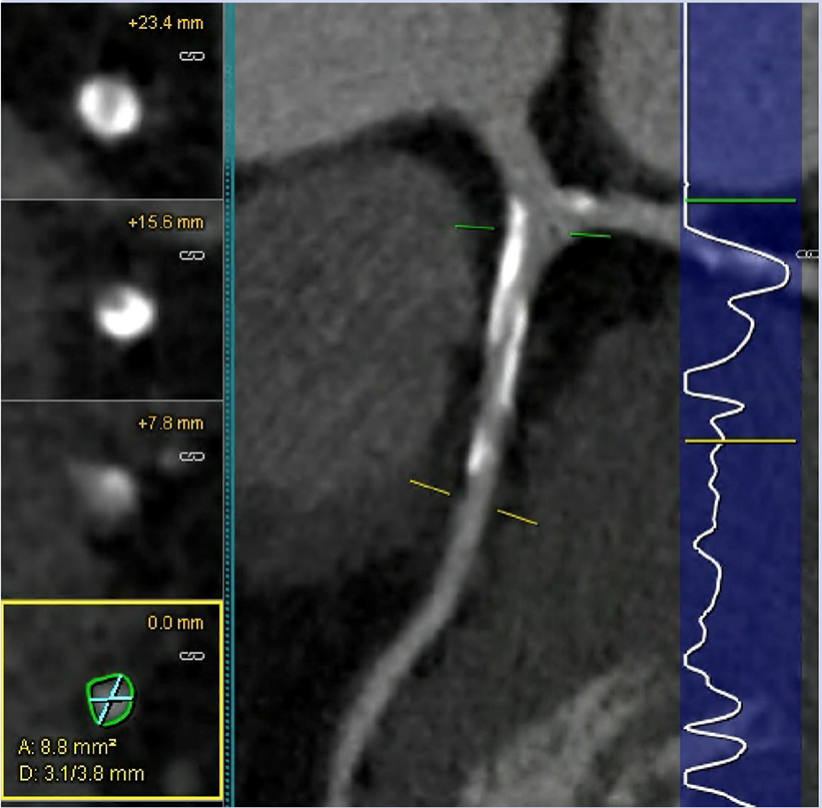
Coronary stenosis	Demonstrated increased risk of MACE with >50% stenosis on CTCA	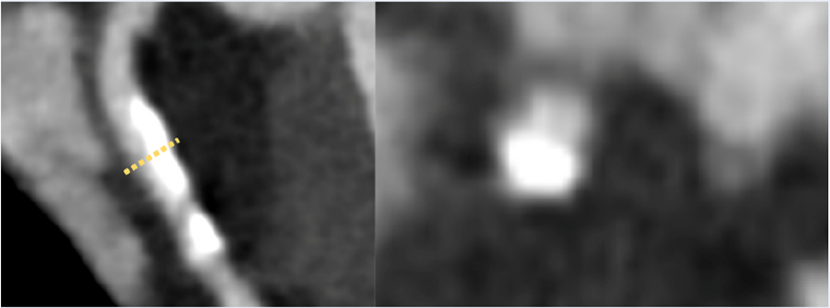
High-risk Plaque Features	– HRP features include spotty calcification, positive remodelling, napkin-ring sign and low attenuation plaque – Low attenuation plaque burden predicts risk of MI – HRP features increased likelihood of MACE independent of significant CAD or clinical assessment	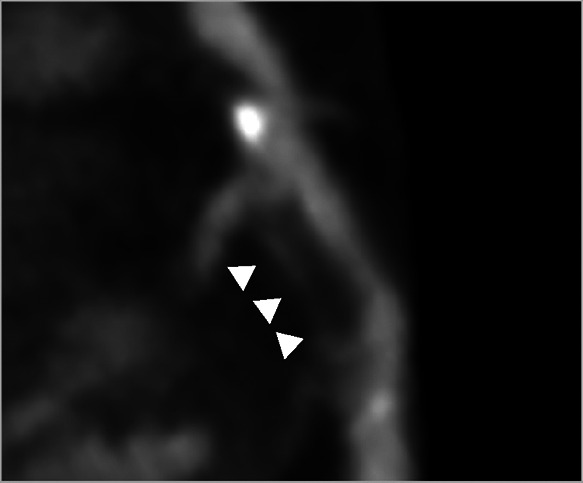 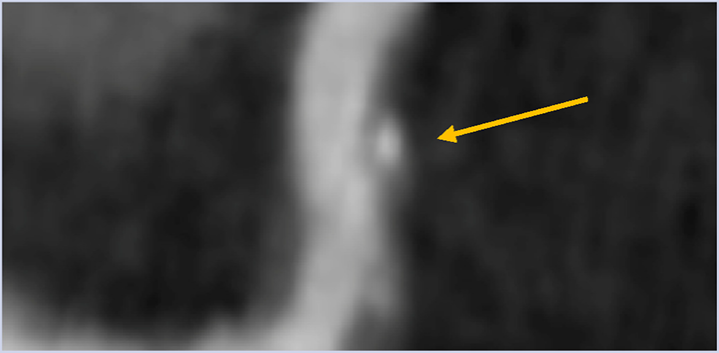
Perivascular Inflammation	Perivascular fat attenuation index enhances cardiac risk prediction and is an indicator of increased cardiac mortality	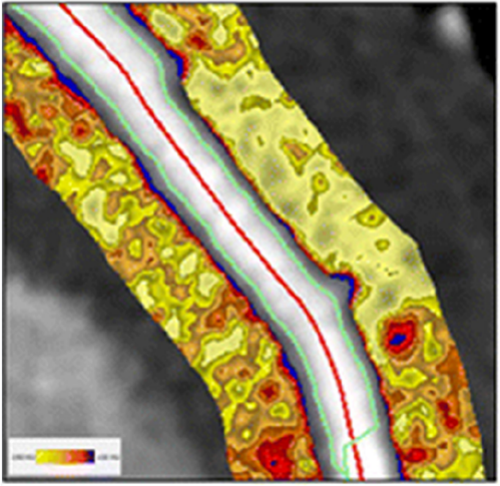

## Optimal trial design: learnings from recent serial coronary CT angiography studies

Amidst the rapid technological advances in both CCTA acquisition hardware and analysis tools, it is important for the field to pause and to consider a harmonised approach in working with regulatory authorities on developing the evidence base for use of CCTA-based measures as valid surrogates for clinical trials. Here, we review the published literature of CCTA-based clinical trials to consider factors such as the specific imaging marker, inclusion criteria, exclusion criteria, sample size, and study length.

### Summary of current evidence in this sphere

There are numerous therapeutic trials using serial CCTA scans with atherosclerosis endpoints reported in the literature (Table 1). We have identified 15 clinical trials which have used CCTA endpoints. Most trials enrolled patients with either consequences of coronary plaque, such as MI, or patients with suspected or known coronary artery disease. Exclusion criteria centred around lack of coronary plaque, patient factors which would limit scan quality or assessment or contraindications to the intended therapy.

Outcome measures selected for each study were consistent across the trials, with standard features of coronary plaque being reported such as total plaque volume, non-calcified volume and high-risk features. CCTA was demonstrated to be able to detect significant interval changes within non-calcified plaque in 12 of the 15 trials. Further positive findings in these trials included changes in total plaque volume, non-calcified plaque percentage (of total plaque) and calcified plaque volume.

The average number of patients included in these trials was 228. It is noted that some of these studies did not perform power calculations or even themselves commented that they were not adequately powered to evaluate clinical endpoints.

The average time to first follow up CT scan was 14.4 months (range 6–24 months). Changes in various coronary CT atherosclerosis measures were observed in all trials, including those with the shortest and longest follow up times.

These trials showed that over a short average period of 14.1 months, within a small average patient number of 228, statistically significant changes to various CCTA outcomes could be demonstrated across a range of therapeutics.

There are associated limitations to the current available evidence. Only two of the trials enrolled participants post-recent MI (27, 33). Both of these trials demonstrated changes in plaque phenotypes due to the intervention, with Vaidya et al. observing decreases in low-attenuation plaque volume in participants on colchicine compared to those on standard of care, independent of high-dose statin therapy. These studies highlight that changes in plaque features post-acute event can be observed utilising CT, however more study in this post-acute event cohort is needed given the small sample sizes.

There is a growing body of evidence for low-attenuation plaque volume as a risk factor for MACE, and this was also used as an outcome measure in several trials (47). However, this is usually a very small proportion of plaque volume in those without advanced active disease, so is harder to measure accurately and may be less reproducible. These additional measures are readily acquired through semi-automated software systems and are therefore appealing to researchers given their ease of collection. High risk plaque features were not demonstrated to reliably change in these included trials which again, likely relates to their small number which made powering for changes in this feature difficult.

This group of studies is subject to publication bias and more data is required, especially patient level trial data, to cement these findings. Larger dedicated studies with all-inclusive CT based endpoints are required in order to assess each individual endpoint's suitability and strength to detect change for application to phase 2 and 3 trials. From the general body of evidence around this subject (Table 1), non-calcified plaque volume is the favoured outcome measure.

### Application to trial design

The incorporation of CCTA-based assessment of coronary plaque changes in clinical trials would require changes in eligibility criteria and consideration of the impact of these changes. First, by definition, participants would need to have CT imaging-identified coronary atherosclerosis in an enrichment trial. In addition, eligibility criteria would need to be considered which address factors affecting image acquisition quality and patient suitability. With the high and increasing use of CCTA in the management of patients with suspected cardiac chest pain, especially in the outpatient setting, selection of patients from this cohort with known coronary plaque would offer a large population from which to recruit. In this setting, patient factors which may reduce image quality—such as the very obese (CT penetration) or very elderly (high calcium burden)—would be efficiently pre-screened. Further assessment of the suitability of proposed intervention in these groups would be done on a trial-by-trial basis depending on the planned intervention.

One potential enrichment opportunity for a robust trial design, would be a high quality CCTA outpatient clinic, such as a rapid access chest pain clinic model. These are commonplace in most large hospital centres and often rely on CCTA as it has an important role in assessment of stable suspected cardiac chest pain. Using the inclusion and exclusion criteria of the previous trials in this space, we have derived the below theoretical ideal trial inclusion and exclusion criteria (Table 3). However, with advances in CT technologies, some of the exclusion criteria may be redefined in the future, as excellent image quality may be now achieved in patients who previously were considered sub-optimal candidates for CCTA.

**Table 3:** Suggestion of possible inclusion and exclusion criteria for trials utilising CCTA endpoints based on based on latest evidence and trials included in Table 1.

Inclusion criteria	Exclusion criteria
Age 18–80 years old Presence of coronary plaque on baseline CCTA	eGFR <30 ml/min/1.73 m^2^ Heavily calcified plaque with bloom artefact or Known CAC score >1,000 AU Previous CABG or stent Poor baseline CT scan quality High body-mass index >3 kg/m^2^

In regards to the proposed inclusion criteria, the age lower limit reflects age of consent for trial involvement. However, in the modern era, it is very unlikely that patients at this age would have coronary disease without a genetic risk, such as familial hypercholesterolaemia. The upper limit of age is less clear, and should include careful considerations of scan quality, disease phenotype, and trial operations (e.g., protocol adherence). The presence of CAD in the elderly is often associated with calcified plaque, increasing the difficulty of luminal assessment. High burden of calcified plaque not only limits scan quality but also is less likely to change over time with therapies and therefore may reduce the significance of outcomes in studies utilising CCTA (50). The study design may benefit from further filtering of inclusion, with the more specific criteria of presence of non-calcified plaque being applied.

Exclusion criteria involve straight forward reasons such as: (a) inability to perform a contrast-related CT study (e.g., severe renal impairment or contrast allergy); and (b) scan quality-related factors. Patients with significant calcified plaque, as discussed above, would have lower quality scans; those with high body-mass index would also have reduced diagnostic accuracy. Patients with these issues were often excluded from the previous studies assessing change in plaque over time as seen in Table 1. Patients with previous CABG typically have severely diseased native vessels and these become calcified over time further limiting assessment. Secondary to the bypass grafts and severe native vessel disease, reduced coronary flow across the diseased coronary segments results in plaque propagation. These patients therefore should be excluded as there are significant variables which would affect plaque progression and regression. Finally, patients with poor quality baseline CT angiograms should be excluded as these are more likely to result in variable plaque estimates which would diminish the accuracy of assessing plaque change at the follow up CT.

Powering these trials would involve understanding of the therapeutic to be studied, its mechanism and animal model data, as well as the baseline atheroma burden of the subjects. The current evidence (Table 1) suggests that a theoretical trial of 318 individuals would have 90% power to detect percentage change in low-attenuation plaque volume by 50% compared to standard of care. This is in line with the percentage change in low-attenuation plaque seen in Noguchi et al. which demonstrated a 76% difference in low-attenuation plaque volume for patients taking statins vs. control (29). Few trials of even this small size of 318 individuals have been done therefore performing trials of 300 or more individuals would be an important next step for this sphere of research.

Selection of primary and secondary outcomes should focus on plaque volume and morphology (Table 4). The value of these outcomes in regards to reduction in risk of MACE has been discussed above and the evidence supporting this is robust. The plaque morphology outcomes which demonstrated the most change in prior trials were changes in non-calcified plaque. Both non-calcified plaque volume (mm^3^) as well as non-calcified plaque percentage (of total plaque) have been shown to be suitable markers for assessment of an interventions therapeutic benefit for phase 2/3 studies (Table 1). Additional outcomes which were able to demonstrate changes in the included serial CT trials, which have also been associated with clinical outcomes previously, are total plaque volume and calcified plaque volume. Further secondary outcomes should include high-risk plaque features (spotty calcification, positive remodelling in respect to remodelling index, napkin-ring sign); however these have not been shown consistently to have statistically significant change over 12-months in the included trials, likely due to lack of power. Calcified plaque volume or calcified plaque as a percentage of total plaque should also be included. Higher levels of calcified plaque is a marker for the presence of more plaque overall and therefore higher risk of MACE, however this is known to increase with statin use and can represent stabilisation of previously non-calcified lesions. Given calcified plaque volume needs to be interpreted in the context of the therapeutic and the clinical situation, collecting more information on this outcome would be important to further our understanding.

**Table 4:** Suggestion of possible primary and secondary outcomes for trials utilising CCTA endpoints based on latest evidence and trials included in Table 1.

Primary outcome	Secondary outcomes
Non-calcified plaque volume (mm^3^)	Total plaque volume (mm^3^) Non-calcified plaque percentage (%) Low-attenuation plaque volume (mm^3^) Calcified plaque volume (mm^3^) High risk plaque features – Low attenuation plaques (no#) – Spotty calcification plaque (no#) – Remodelling index (RI)

Standardising CT protocols across multiple sites with differing scanners presents a unique challenge (51). There are multiple steps within a CT protocol that would need to be standardised including slice thickness, voltage, rotation time, and R-R interval this is easily protocolised across sites with modern CT scanners. To minimise step artefact and to increase CT accuracy, trial sites should be selected with dual-source, wide-detector scanners, which can perform whole heart single rotation acquisition. In the not-to-distant future, studies could be performed on CT scanners using photon-counting detectors to improve spatial resolution and iodine signal. However, these are not yet widely available commercially and their use in phase 2 clinical trials would currently be challenging.

Timeline of serial CT assessment of plaque morphology changes should include careful consideration on the mechanism of action of the therapeutic intervention. Applying the lessons learned from previous trials, a timeframe of 12–18 months for serial CCTA assessment is reasonable to assess for alterations in plaque volume and morphology.

Plaque assessment is typical performed initially by semi-automated AI based programs (vendor specific). The highest accuracy is then achieved with expert reporter correction of the AI calculation (52). Standardisation of the program used for plaque assessment would be important for reproducibility and should be agreed upon by the study co-ordinator and peripheral sites at the outset. Correlation between expert interpretation has been overall excellent across multiple studies including those listed above (Table 1). Collating CT data from multiple sites within one core lab with central quality control of image acquisition would not present an issue with reproducibility and would allow increased patient recruitment and follow up given the availability of multiple sites. Use of a single core laboratory to measure scans is essential to maximising measurement quality and reproducibility. There is excellent reproducibility for non-calcified plaque volume, calcified plaque volume and total plaque volume as well as modest reproducibility for low attenuation plaque volume as it is much smaller (53, 54).

The complex operational considerations for including CCTA in clinical trials is summarised below (Table 5).

**Table 5:** Operational considerations for including CCTA in clinical trials. Description of trial components and the trial design considerations associated with these components.

Trial component	Design considerations
Study leadership and organization	• Investigator(s) with CTA trial experience on executive committee • CCTA core lab • Image deidentification, transmission and management
CCTA endpoints	• Determination of primary and secondary CTA endpoints including specification of per lesion, per vessel and per person analysis
Inclusion/exclusion criteria	• Site vs. core lab determined
Study documents	• Manual of operations for image acquisition and transmission • Core lab charter for image analysis and storage
Site qualification	• Personnel and training • Test image acquisition and transmission
Study conduct	• Real time central monitoring of site timeliness and image quality • Periodic re-training and remediations as needed
Core lab image analysis	• Real time central monitoring of analysis timeliness and quality • Periodic re-training and remediations as needed
Statistical analysis	• Develop SAP for image analysis, including CCTA investigators • Strategies to deal with missing/noninterpretable scans and site-read qualifying scans that do not qualify by core lab read
Results dissemination	• Inclusion of CTA investigators, core lab PI as an author on any paper reporting imaging results

## Coronary CT angiography limitations for consideration in study design

As with any imaging modality, CCTA has unique limitations, which largely centre around patient factors and scanner factors. Patient factors include the presence of coronary stents and coronary calcium. Coronary stent CT imaging can encounter issues including current spatial resolution and partial voluming. Yan, et al. demonstrated that patients with previous percutaneous coronary intervention (PCI) were more likely to have false positive diagnosis of obstructive CAD on CT (55). As previously described, coronary calcium causes blooming artefact which can lead to over, or in some circumstances under, estimation of stenosis. Yan, et al. again demonstrated that patients with higher absolute coronary calcium score were more likely to have a false positive diagnosis of obstructive disease. This has been reduced by recent advancements in CT technology, such as decreased voxel volume which reduces the partial volume effect of calcium. Pack, et al. discussed multiple available techniques to reduce CT calcium artefact including both scanner technology and image processing. Further CT developments have led to the availability of new photon-counting CT detectors, which have much higher spatial resolution to reduce the root cause of CT calcium artefact which is partial volume. Although current access to photon-counting CT is limited, sites with this capacity could begin to be prioritised as trial recruitment sites, providing further inventive for investment by institutions in this technology. As with all new technology, it is predicted that both the cost and availability of these scanners will reduce with time.

CT technology comes with radiation exposure, which is related to cancer in a dose-dependent manner. Adoption of the “as low as reasonably achievable” (ALARA) methodology in all centres and general improvements in CT technology has resulted in dramatic reductions in radiation from CT over the past decade. The multi-national PROTECTION-IV study demonstrated that CCTA studies performed in 2017 had an average dose of 252 mGy*cm [interquartile range (IQR) 154–412] (56). This represented a 78% reduction in exposure from PROTECTION-I in 2007 (dose length product for CCTA portion only: 885 mGy*cm). Further studies into the new photon-counting CT technologies are needed to assess the average radiation dose. However, early emerging data seems to suggest a likely reduction in radiation dose by up to 30% (57). Based on the interquartile range from PROTECTION-IV, this is the equivalent of 2–5.6 mSv effective dose. With low levels of radiation exposure and continuing improvements in effective radiation dose to patients, the harm of repeated scanning is substantially reduced and therefore unlikely to be an issue in trial ethics.

## Next steps

Current evidence gaps need to be closed in order to further the use of CCTA as a clinical trial endpoint. Meta-analyses of available clinical trial and cohort study data—including both trial-level and individual patient-level—with imaging components should be performed, to strengthen the association between changes in CT-based coronary atherosclerosis measures and reduction in risk of MACE. In particular, the relation of non-calcified plaque volume, high-risk plaque features, and how the change of these coronary findings over time associates to differences in risk of MACE. If positive, this data would strengthen the use of CCTA-based measures in risk assessment, potentially serving as an early enrichment tool in future pharmaceutical clinical trials. The ideal application of CCTA to cardiovascular outcome trials would need to be further analysed with subsequent research in this area focused on the ideal protocol design, sample size calculation, sampling frequency and image acquisition and analytic strategies. Establishing a benchmark clinical trial design would assist the field in more efficient translation of therapeutic candidates into human study.

Using these steps, a formal submission to regulatory bodies could be made which would unlock the use of CT outcome use in clinical trials. Ongoing evaluation of new data from an increased use of serial CCTA would then lead to an explosion of evidence in this space, further refining both the protocol designs and application of serial CT in coronary disease. The CAD Frontiers ACTION A2D2 group is actively working on a path forward for the coupling of CCTA based outcomes and clinical trials.

## Conclusion

CCTA provides a fast non-invasive assessment of coronary plaque burden and is, by extension, an attractive option for the assessment of CAD therapeutic efficacy. CCTA outcomes are closely linked with MACE and have been used widely to predict cardiovascular risk. Studies using CCTA-based measures as outcomes would need to carefully consider patient and technical-based limitations of the CT technology. However, the efficiency gained has the opportunity to reduce sample size and longitudinal follow-up for early trials, providing an easier and less costly pathway to clinical development of CAD therapeutics. To progress forward, trial data utilising CCTA outcomes is needed to present to regulatory bodies a robust case for the use of these outcomes to enrich phase 2 and 3 clinical trials.
